# GC×GC-TOFMS Analysis of Essential Oils Composition from Leaves, Twigs and Seeds of *Cinnamomum camphora* L. Presl and Their Insecticidal and Repellent Activities

**DOI:** 10.3390/molecules21040423

**Published:** 2016-03-28

**Authors:** Hao Jiang, Jin Wang, Li Song, Xianshuang Cao, Xi Yao, Feng Tang, Yongde Yue

**Affiliations:** 1 College of Plant Protection, Anhui Agricultural University, Hefei 230036, China; ahjh88@163.com; 2 SFA Key Laboratory of Bamboo and Rattan Science and Technology, International Centre for Bamboo and Rattan, No. 8 Futong Dongdajie, Wangjing, Chaoyang District, Beijing 100102, China; songliicbr@163.com (L.S.); caoxianshuang123@163.com (X.C.); yaoxi@icbr.ac.cn (X.Y.); fengtang@icbr.ac.cn (F.T.); 3 State Key Laboratory of Tea Plant Biology and Utilization, Anhui Agricultural University, Hefei 230036, China

**Keywords:** *Cinnamomum camphora*, essential oils, GC×GC-TOFMS, contact toxicity, cotton aphid, repellent activity, linalool

## Abstract

Interest in essential oils with pesticidal activity against insects and pests is growing. In this study, essential oils from different parts (leaves, twigs and seeds) of *Cinnamomum camphora* L. Presl were investigated for their chemical composition, and insecticidal and repellent activities against the cotton aphid. The essential oils, obtained by hydrodistillation, were analyzed by GC×GC-TOFMS. A total of 96 components were identified in the essential oils and the main constituents found in the leaves and twigs were camphor, eucalyptol, linalool and 3,7-dimethyl-1,3,7-octatriene. The major components found in the seeds were eucalyptol (20.90%), methyleugenol (19.98%), linalool (14.66%) and camphor (5.5%). In the contact toxicity assay, the three essential oils of leaves, twigs and seeds exhibited a strong insecticidal activity against cotton aphids with LC_50_ values of 245.79, 274.99 and 146.78 mg/L (after 48 h of treatment), respectively. In the repellent assay, the highest repellent rate (89.86%) was found in the seed essential oil at the concentration of 20 μL/mL after 24 h of treatment. Linalool was found to be a significant contributor to the insecticidal and repellent activities. The results indicate that the essential oils of *C. camphora* might have the potential to be developed into a natural insecticide or repellent for controlling cotton aphids.

## 1. Introduction

As an alternative to toxic pesticides, essential oils have attracted particular attention because of their specificity to pests, their biodegradable nature and their potential for commercial application [1]. *Cinnamomum camphora* (L.) Presl (family: Lauraceae), commonly known as the camphor tree, is a large evergreen tree and is widely distributed in subtropical zones, including southeastern China and northeastern Australia [2]. *C. camphora* has long been recognized as a source of essential oil. The essential oil of *C. camphora* can be utilized as a medicine and perfume. According to the previous studies, the essential oil from *C. camphora* has various bioactive properties, such as antioxidant [3], antibacterial [4,5], antifungal [6], insecticidal [1,7] and repellent activities [1]. The leaves and bark of *C. camphora* are rich in terpenoids, sesquiterpenes and phenylpropanoids, which are an important group of secondary metabolites and are associated with these bioactivities [6,8].

The cotton aphid (*Aphis gossypii* Glover) is one of the most serious pests of cotton throughout the world [9]. It can cause damage to the host plant not only by direct feeding, but also through transmission of viral diseases [10]. At present, chemical pesticides are still the primary method for controlling aphids on crop plants [11]. However, the indiscriminate use of these chemical pesticides is very harmful for human health and the environment [12]. It is well known that secondary metabolites of some plants may act as insecticides, such as flavonoids (rotenone) [13], terpenes (azadirachtin) [14,15], alkaloids (oxymatrine) [16] and essential oils [17,18,19,20]. Therefore, botanical pesticides have been considered as an attractive alternative to chemical pesticides. To the best of our knowledge, there is still no reported work on the insecticidal and repellent activities against cotton aphids from the essential oils of *C. camphora.*

Traditionally, the chemical composition of *C. camphora* essential oil has been detected by GC and GC-MS. Compared with the traditional GC, two-dimensional GC (2D-GC) technology is an emerging technology that provides higher peak capacity, greater separation capacity and an improved signal-to-noise ratio [21]. Comprehensive two-dimensional GC coupled to time-of-flight mass spectrometry (GC×GC-TOFMS) is a powerful technology, which has been successfully applied for qualitative and quantitative analysis of the chemical composition of different plants [22,23].

Therefore, the aims of this study were to investigate the chemical composition of the essential oils from different parts of *C. camphora* using GC×GC-TOFMS, and to evaluate the insecticidal and repellent activities of the essential oils against cotton aphids.

## 2. Results and Discussion

### 2.1. Chemical Composition of the C. Camphora Essential Oils

The yields of leaf, twig and seed essential oils obtained by water distillation were 0.86% (*w*/*w* relative to dry material weight), 0.48% and 2.2%, respectively. In order to obtain higher separation efficiency, GC×GC-TOFMS was used to analyze the essential oils of *C. camphora*. The identification of compounds was carried out by comparing the mass spectra obtained with those from the NIST2011 mass spectral library or with mass spectra from the literature [22,24,25,26], and by co-injection of available standard compounds, such as eucalyptol (purity 99%), camphor (purity 96%), methyleugenol (purity ≥ 98%) and linalool (purity 97%). Tentative identification of some terpenoids was carried out using retention indices (RI), the zone of elution and mass spectra. The peaks with matching similarity of more than 80% were accepted as candidate compounds. The quantification was carried out by peak area normalization. The GC×GC-TOFMS analysis results for the oils are presented in Table 1.

As seen in Table 1, a total of 96 compounds were identified in leaf, twig and seed essential oils. Among these compounds, 67 compounds were identified in the leaf essential oil of *C. camphora*, representing 92.33% of the total oil, and the major compounds were camphor (18.48%), eucalyptol (16.46%), linalool (11.58%) and 3,7-dimethyl-1,3,7-octatriene (11.07%). A total of 79 identified constituents which could account for 90.57% of the total essential oil from *C. camphora* twigs, in which the main compounds were eucalyptol (17.21%), camphor (13.17%) and 3,7-dimethyl-1,3,7-octatriene (11.47%), were identified. Sixty components constituted 94.98% of the seed oil. The major components were eucalyptol (20.90%), methyleugenol (19.98%), linalool (14.66%) and camphor (5.5%).

The chemical composition of *C. camphora* leaf and twig oils was similar. Fifty-nine compounds were common, among which the main components were camphor, eucalyptol, 3,7-dimethyl-1,3,7-octatriene, linalool and terpineol. At present, no data was reported on the chemical composition of the essential oil of *C. camphora* seed. In contrast to the leaf and twig oils, the seed oil was characterized by a high content of methyleugenol (19.98%). According to the previous studies, *C. camphora* could be divided into five chemical types by the main compounds of its leaf oils, such as camphor-type, linalool-type, cineol-type, isonerolidol-type and borneol-type [7]. Therefore, the tested *C. camphora* sample probably belonged to the camphor-type because it contained rich camphor.

The composition of the essential oils of *Cinnamomum* species has been widely investigated and the main components were similar. The *Cinnamomum* species oils were found to mainly contain linalool, eucalyptol, camphor, terpinen-4-ol, limonene, terpineol and safrole [27,28,29,30]. The major compounds from *C. camphora* oils were similar to the previous reports. However, the relative amounts (based on the peak areas) of the individual components were different. For example, in this study the content of camphor in the essential oil of *C. camphora* leaf was 18.48% while the content of camphor in the essential oil of *C. camphora* leaf collected from India was 67.23% [31]. These differences in chemical compounds of the essential oils could be due to several factors such as harvest time, local climate, extraction method and varieties.

### 2.2. Contact Toxicity

The essential oils from *C. camphora* leaves, twigs and seeds showed strong contact toxicity against cotton aphids with median lethal concentration (LC_50_) values of 245.79, 274.99 and 146.78 mg/L after 48 h of treatment, respectively (Table 2).

**Figure 1:**
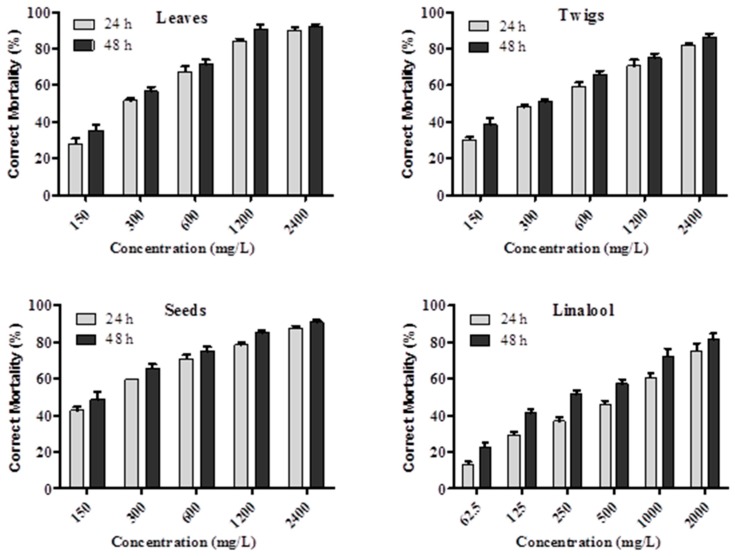
Contact toxicity of *C. camphora* essential oils and linalool on cotton aphids at different concentrations after 24 h and 48 h of treatment. Error bar represents the standard deviation of the mean (*n* = 3).

As seen from Table 2, the essential oil of *C. camphora* seeds (LC_50_ = 146.78 mg/L) showed higher contact toxicity against cotton aphids than that of *C. camphora* leaves (LC_50_ = 245.79 mg/L) and twigs (LC_50_ = 274.99 mg/L). Compared with the commercial insecticide imidacloprid (LC_50_ = 3.58 mg/L), the essential oil of *C. camphora* seeds demonstrated 41 times less toxicity after 48 h of treatment. The imidacloprid also showed acute contact toxicity to cotton aphids with an LC_50_ value of 12.53 mg/L after 24 h of treatment. However, compared with the other plant essential oils in the published reports, for example essential oils of *Cynanchum mongolicum* (LC_50_ = 38.4 μL/mL, after 48 h of treatment) and essential oils of *Rosmarinus officinalis*, *Schinus areira* and *Tagetes terniflora* (LC_50_ = 15.2, 58.3, and 76.2 mg/mL after 24 h of treatment, respectively) [32,33], the essential oil of *C. camphora* seeds revealed a stronger level of contact toxicity against cotton aphids. In Figure 1, it can be seen that the contact toxicity of essential oils from *C. camphora* and linalool against cotton aphids was concentration-dependent.

Linalool, a monoterpene alcohol, has been proved to possess insecticidal activities [34]. For example, linalool isolated from *Zanthoxylum schinifolium* essential oils exhibited contact activity against *Sitophilus zeamais* with an LD_50_ value of 13.90 μg/adult [35]. In this study, linalool also exhibited contact toxicity against cotton aphids with an LC_50_ value of 262.77 mg/L after 48 h of treatment. Therefore, linalool could be one of the active compounds in the essential oils of *C. camphora*. Additionally, the major compounds from *C. camphora* oils such as eucalyptol, terpineol, caryophyllene and limonene also exhibited stronger contact toxicity against various pests [36,37,38].

### 2.3. Repellent Activity

The repellent activity of the essential oils of *C. camphora* against cotton aphids was evaluated. The results are presented in Table 3.

As shown in Table 3, the essential oil of *C. camphora* seeds was more effective compared to the essential oils of *C. camphora* leaves and twigs in terms of repellent rate, but it was less effective than linalool at the concentration of 10 µL/mL. At the tested concentrations, the positive control, DEET, exhibited strong repellency at 12 h after treatment. Compared with the positive control, only at the highest concentration of 20 µL/mL, the repellent rate (76.19%) of the seed oil of *C. camphora* was almost the same as that of DEET (repellent rate = 77.42%, tested at the concentration of 5 µL/mL). Therefore, a higher concentration (more than 10 µL/mL) is recommended when *C. camphora* essential oils are used as a repellent.

Meanwhile, many essential oils have been evaluated for repellency against insects mentioned in the literature. For example, in a certain range of concentration (1.25–10.0 µL/mL), the essential oil from *C. mongolicum* showed high repellent activity against soybean aphids (*Aphis glycines*) at 2 h exposure [32]. The essential oil of *Tagetes terniflora* and *Schinus areira* leaves exhibited repellency against aphids with a repellent index of 66.66% and 73.33% at 24 h exposure, respectively [33]. The essential oils of *Rosmarinus officinalis* L. and *Mentha spicata* L. showed a strong repellency effect against *Ixodes ricinus* Nymphs in the laboratory bioassay. Furthermore, *R. officinalis* and *M. spicata* exhibited good repellency (68.29% and 59.38%, respectively) in the field trial [39].

In general, the insecticidal and repellent activity of essential oils of could not be easily correlated with one specific compound. In this study, linalool also showed strong repellent activity against cotton aphids with a repellent rate in the range of 37.50% to 76.56% at the given concentrations after 24 h of treatment. Therefore, as one of the major components in the essential oils of *C. camphora*, linalool could be one of the active compounds.

Above all, this is the first report that the *C. camphora* essential oils showed strong insecticidal and repellent activities against cotton aphids. Linalool may be one of the important activity components, as it was proved to possess insecticidal and repellent activity against cotton aphids. The essential oils of *C. camphora* could be potential alternatives to the traditional chemical control of cotton aphids. For the practical use of *C. camphora* oils and their constituents as novel insecticides, further studies on the insecticidal and repellent mechanisms and safety evaluations of *C. camphora* essential oils are needed. Additionally, the characteristic components (such as methyl eugenol, eucalyptol and camphor) are needed to evaluate their contribution to the insecticidal and repellent activities.

## 3. Materials and Methods

### 3.1. Plant Material

The leaves, twigs and seeds of *C. camphora* were collected in Nanchang, Jiangxi Province, China on 25 September 2014. The *C. camphora* trees were cultivated in the garden. A total of nine trees were randomly selected for samples collection (650 g of seeds from nine trees; 3080 g of leaves collected from each tree; 2560 g of small twigs collected from each tree). The plant material was authenticated by Prof. Yimin Hu from Anhui Academy of Forestry, China. The samples were dried in the shade and ground into powder, and stored at −20 °C.

### 3.2. Chemicals

The HPLC-grade methanol and hexane were purchased from fisher scientific (Fair Lawn, NJ, USA). Analytical-grade chemical was obtained from Beijing Chemical Works (China). Water was purified with an ultrapure water system (Purelab Plus, Pall, Port Washington, NY, USA). The standard compound of imidacloprid (purity, 96%, a commercial aphidicide) was obtained from National Pesticide Quality Supervision and Inspection Centre (Beijing, China). *N,N*-Diethyl-3-methylbenzamide (DEET), eucalyptol (purity 99%), camphor (purity 96%), methyleugenol (purity ≥98%) and linalool (purity 97%) were purchased from Sigma-Aldrich (St. Louis, MO, USA). Alkanes (C_7_–C_30_) was purchased from Supelco (Bellefonte, PA, USA). All other analytical-grade chemicals were obtained from Beijing Chemical Works (Beijing, China).

### 3.3. Insect

A continuous culture of *A. gossypii* is maintained in a temperature-controlled incubator at 28 ± 1 °C, 70% ± 5% relative humidity (RH) and exposed to a long photoperiod (16 h light:8 h dark, 16:8 LD) in the laboratory. The cotton aphids were reared on the leaf blades of cotton seedlings, and all bioassays were carried out using apterous adult aphids.

### 3.4. Essential Oils

Essential oils were prepared according to the method described in previous studies [6,7] with a slight modification. The dried plant powder (50 g) was subjected to hydrodistillation in a Clevenger-type apparatus for six hours. The obtained essential oils were dried by anhydrous sodium sulphate (Na_2_SO_4_). The essential oils were accurately weighed. All experiments were carried out in triplicate. The oil yields were calculated on the basis of the dry weight of plant material. The formula was as follows:

(1) Essential oils yield (%) = W_1_/W_2_ × 100

where W_1_ was the net weight of oils (grams) and W_2_ was the total weight of dry samples (grams).

### 3.5. GC×GC-TOFMS Analysis

The essential oil samples from three parts (leaves, twigs and seeds) of *C. camphora* (10 μL) were diluted with *n*-hexane (1 mL), respectively. The GC×GC-TOF/MS analysis was carried out on an Agilent 7890B gas chromatography system (Agilent Technologies, Santa Clara, CA, USA), equipped with a Pegasus 4D TOFMS (LECO Corporation, St. Joseph, MI, USA). The GC×GC system contained two chromatography columns. The first column was a nonpolar Rxi-5 Sil MS (5% phenyl-95% dimethyl arylene polysiloxane, 30 m × 0.25 mm i.d. × 0.25 μm film thickness) and the second column was a medium polarity Rxi-17 Sil MS with dimensions of 2 m × 0.18 mm i.d. × 0.18 μm film thickness, 50% phenyl-50% dimethyl arylene polysiloxane (Restek Corp., Bellefonte, PA, USA). Helium was used as a carrier gas at a constant flow rate of 1 mL/min. The initial temperature of the first column was set at 50 °C for 12 s and then ramped to 280 °C at a rate of 8 °C/min. The secondary oven was set at a 5 °C offset above the primary oven. The modulator temperature offset and transfer line temperature were 15 °C and 280 °C, respectively. The modulation period was 4 s and the hot pulse was set at 0.8 s. The injection volume was 1 μL in split mode, at a ratio of 50:1. The temperature of the injector was kept at 240 °C. The mass spectrometer was set to scan in the range of *m*/*z* 33–550 at an acquisition rate of 100 spectra/s. The detector voltage was set at 1420 V, and the electron energy was set to 70 eV. The ion source temperature was kept at 250 °C. All the operations and analysis of data were controlled using LECO ChromaTOF software version 4.52.

### 3.6. Contact Toxicity Bioassay

The contact toxicity was measured using topical application method as previously described [10,40,41] and with a slight modification. In each test, a total of 50 aphids were selected on clean cotton seedlings. Sample solutions were deposited on the dorsum of the thorax of each aphid by an auto micro-applicator (900-X, Burkard Manufacturing Co. Ltd., Rickmansworth, UK). Based on the preliminary screening result, the essential oils and standard chemicals were serially diluted in acetone to evaluate the dose-effect relationships against cotton aphid. Each essential oil was diluted into different concentrations (150, 300, 600, 1200 and 2400 µg/mL). Imidacloprid, as a positive control, was diluted into five concentrations (1, 5, 10, 50 and 100 µg/mL). Linalool was diluted into six concentrations (62.5, 125, 250, 500, 1000 and 2000 µg/mL). Control aphids were treated only with acetone. Each treatment was replicated three times. After application, the cotton seedlings with treated aphids were transported to petri dishes (9.0 cm i.d. × 1.5 cm) and maintained at controlled temperature (28 ± 1 °C) and humidity (70% ± 5%) conditions in the light incubator (16:8 LD). Aphid mortality was assessed 24 h and 48 h after application. The corrected mortality rates were measured using Abbott’s formula [42].

(2) Correct mortality (%) = (M_1_ − M_c_)/(100 − M_c_ ) × 100

where M_1_ (%) was the mortality of the treated groups and Mc (%) was the mortality of the control groups.

### 3.7. Repellent Activity Bioassay

**Figure 2:**
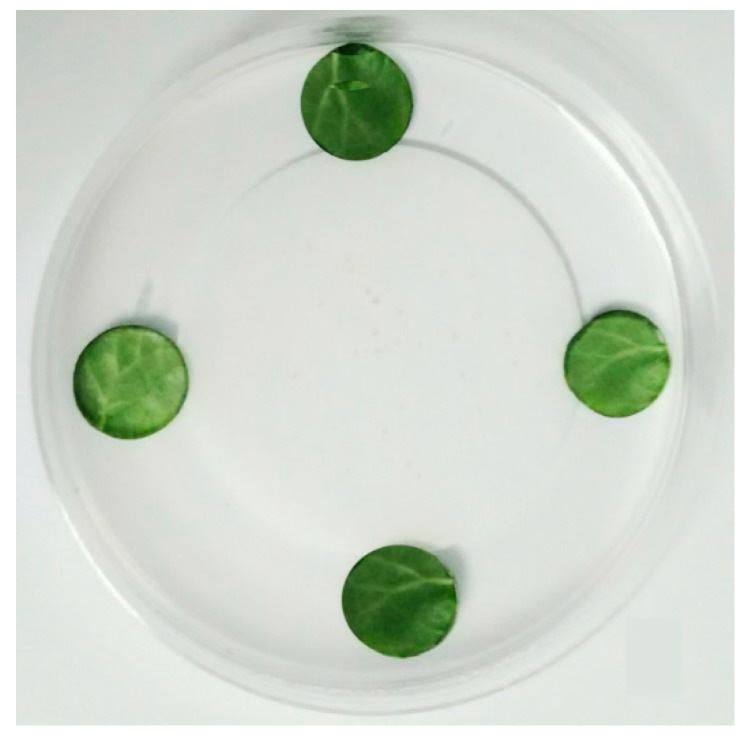
Layout of leaf discs of cotton in the Petri dish.

Repellent activity bioassay was used to assess behavioral response of cotton aphid to essential oil volatiles. The repellency of *C. camphora* essential oils against cotton aphids was determined as previously described and with a slight modification [43,44]. The essential oils were diluted in ethanol to serial concentrations (1, 2, 5, 10 and 20 µL/mL). A commercial repellent, DEET, as a positive control, was diluted to six concentrations (0.01, 0.1, 0.5, 2, 5 and 10 µL/mL). Linalool was diluted to five concentrations (0.5, 1, 2, 5 and 10 µL/mL). Fresh leaf discs of cotton (1.5 cm in diameter) were dipped into the prepared sample solutions for 5 s. The leaf discs were air dried in fuming hood and then transferred to the Petri dishes (9.0 cm in diameter, with 2% solidified agar beds and filter paper). Leaf discs soaked identically in ethanol served as the controls. The filter paper (6.5 cm in diameter, Whatman No. 2) was placed in the center of Petri plates (Figure 2).

Thirty cotton aphids were released at the center of each filter paper disc and the lid was sealed in place with parafilm. Petri dishes were maintained at 28 ± 1 °C and 70% ± 5% RH with a 16:8 h light:dark photocycle in a light incubator. Three replicates were used for each concentration, so that a total number of 90 cotton aphids were tested at each concentration.

Aphids were calculated to move on the cotton leaf after 12 h and 24 h. The repellent rate (Rr) of each essential oil/compound was calculated using the formula

(3) Repellent rate (Rr) (%) = (N_C_ − N_T_)/(N_c_ ) × 100

where N_C_ was the number of cotton aphids on the leaf disc in the negative control groups and N_T_ was the number of cotton aphids on the leaf disc in the treated groups.

### 3.8. Statistical Analysis

Statistical significance was carried out applying one-way ANOVA followed by Duncan’s test at *p* = 0.05, using SPSS version 19.0 (IBM Corp., Armonk, NY, USA). Median Lethal Concentration (LC_50_ with 95% confidence intervals) is expressed in milligrams of the material per liter. Probit analysis of concentration and aphid mortality data was performed to evaluate the LC_50_ value [35].

## 4. Conclusions

The GC×GC-TOFMS analysis results revealed that camphor, eucalyptol, linalool, 3,7-dimethyl-1,3,7-octatriene and methyleugenol were the major components in the essential oils of *C. camphora.* Linalool has proved to be a significant contributor to the insecticidal and repellent activities against cotton aphids. This study indicates that the essential oils of *C. camphora* seeds possess potent insecticidal and repellent activities. *C. camphora* can be a promising source of natural insecticide or repellent for controlling cotton aphids.

## Figures and Tables

**Table 1:** Chemical compounds identified in the essential oils from *C. camphora* by two-dimensional gas chromatography coupled to time-of-flight mass spectrometry (GC×GC-TOFMS).

No.	Compound		RI	Rt (s) ^1^	Peak Area (%)		
					Leaf	Twig	Seed
1	methyl isobutyl ketone		<800	196, 0.98	0.04	0.04	Tr ^2^
2	hexanal		<800	236, 1.19	0.03	0.02	- ^3^
3	3-hexen-1-ol		843	284, 1.33	0.03	tr	-
4	(*E*)-2-hexenal		843	284, 1.48	0.03	-	-
5	1-hexanol		858	296, 1.25	0.03	-	-
6	α-thujene		924	356, 1.10	0.11	0.10	0.27
7	α-pinene		935	368, 1.14	1.20	0.76	1.00
8	camphene		950	384, 1.23	0.41	0.33	0.23
9	sabinene		973	408, 1.30	1.95	0.68	1.36
10	β-pinene		980	416, 1.31	1.25	0.51	1.54
11	α-phellandrene		1006	444, 1.33	0.09	0.14	2.70
12	α-terpinene		1017	456, 1.34	0.19	0.21	0.83
13	*p*-cymene		1024	464, 1.54	0.16	0.51	1.10
14	limonene		1028	468, 1.38	0.92	0.65	2.32
15	eucalyptol		1034	476, 1.54	16.46	17.21	20.90
16	β-ocimene		1042	484, 1.36	tr	0.16	0.21
17	γ-terpinene		1056	500, 1.47	0.51	0.98	1.94
18	terpinolene		1084	532, 1.53	0.15	0.23	0.44
19	*trans*-linalool oxide		1084	532, 1.65	0.12	0.18	tr
20	3,7-dimethyl-1,3,7-octatriene		1095	544, 1.67	11.07	11.47	-
21	linalool		1099	548, 1.57	11.58	5.13	14.66
22	hotrienol		1099	548, 1.68	0.59	0.79	-
23	6-methyl-3,5-heptadiene-2-one		1099	548, 2.12	-	0.03	-
24	*E*,*E*-2,6-dimethyl-1,3,5,7-octatetraene		1127	580, 1.62	0.06	0.03	-
25	camphor		1149	604, 2.20	18.48	13.17	5.55
26	octanoic acid		1160	616, 1.54	-	-	0.03
27	*endo*-borneol		1174	632, 1.90	0.30	0.16	-
28	terpinen-4-ol		1181	640, 1.83	0.87	1.11	1.01
29	α-terpineol		1196	656, 1.90	5.00	4.38	2.98
30	estragole		1196	656, 2.16	-	-	0.04
31	*E,E*-2,6-dimethyl-3,5,7-octatriene-2-ol		1203	664, 1.86	0.08	-	-
32	*trans*-3-methyl-6-(1-methylethyl)-2-cyclohexen-1-ol		1207	668, 1.87	-	0.04	0.03
33	carveol		1215	676, 2.09	0.06	0.04	-
34	citronellol		1219	680, 1.67	0.02	0.02	-
35	(*Z*)-3,7-dimethyl-2,6-octadienal		1234	696, 2.02	0.03	0.02	-
36	(*Z*)-3,7-dimethyl-2,6-octadien-1-ol		1245	708, 1.83	0.04	0.09	0.07
37	(*E*)-3,7-dimethyl-2,6-octadienal		1264	728, 2.04	0.03	0.02	-
38	bornyl acetate		1283	748, 1.84	0.25	0.25	0.03
39	safrole		1290	756, 2.42	0.04	0.05	3.28
40	thymol		1294	760, 2.20	-	0.03	-
41	δ-elemene		1337	804, 1.45	0.07	-	0.08
42	α-cubebene		1349	816, 1.45	0.04	0.17	tr
43	2-methoxy-3-(2-propenyl)-phenol		1349	816, 2.47	-	0.13	-
44	*n*-decanoic acid		1361	828, 1.65	-	-	1.72
45	neryl acetate		1369	836, 1.80	-	-	0.03
46	unknown		1369	836, 1.82	0.02	tr	-
47	ylangene		1373	840, 1.52	0.08	0.08	0.07
48	α-copaene		1381	848, 1.51	0.18	0.96	0.35
49	β-elemene		1389	856, 1.58	0.42	0.38	0.31
50	methyleugenol		1393	860, 2.55	0.12	0.40	19.98
51	dodecanal		1401	868, 1.58	-	0.04	0.04
52	α-gurjunene		1414	880, 1.60	0.04	0.21	0.24
53	α-santalene		1418	884, 1.52	-	0.12	-
54	β-caryophyllene		1426	892, 1.69	3.40	3.13	1.71
55	γ-elemene		1435	900, 1.49	-	0.03	-
56	β-famesene		1447	912, 1.48	-	0.03	-
57	aromandendrene		1447	912, 1.66	0.34	0.80	0.38
58	β-santalene		1460	924, 1.62	-	0.04	-
59	α-humulene		1464	928, 1.75	2.62	4.12	1.23
60	γ-gurjunene		1473	936, 1.83	0.08	-	-
61	γ-muurolene		1477	940, 1.72	0.29	1.24	0.54
62	1-(1,3-dimethyl-3-cyclohexen-1-yl)-ethanone		1481	944, 1.80	-	0.02	-
63	germacrene D		1485	948, 1.79	3.76	0.46	0.57
64	α-selinene		1494	956, 1.79	-	6.13	2.87
65	α-muurolene		1502	964, 1.79	1.91	0.29	0.48
66	β-bisabolene		1507	968, 1.59	-	0.04	-
67	δ-cadinene		1520	980, 1.79	0.45	3.42	0.56
68	*trans*-calamenene		1524	984, 1.98	0.07	0.68	0.04
69	cadina-1(2),4-diene		1537	996, 1.82	0.07	0.57	0.07
70	1,2,3-trimethoxy-5-(2-propenyl)-benzene		1537	996, 2.68	-	-	0.06
71	(*E*)-α-bisabolene		1542	1000, 1.87	0.07	0.67	-
72	α-calacorene		1546	1004, 2.13	tr	0.08	0.02
73	unknown		1551	1008, 1.61	-	-	0.16
74	selina-3,7(11)-diene		1551	1008, 1.81	-	-	0.12
75	(*E*)-nerolidol		1555	1012, 1.70	2.13	-	0.22
76	unknown		1569	1024, 1.94	0.57	0.48	0.16
77	unknown		1577	1032, 1.48	-	-	0.09
78	spathulenol		1581	1036, 2.15	0.53	0.76	0.10
79	unknown		1586	1040, 1.85	0.10	0.06	-
80	gleenol		1586	1040, 1.89	-	0.07	-
81	caryophyllene oxide		1591	1044, 2.16	0.39	1.38	-
82	β-elemenone		1595	1048, 1.99	-	-	0.08
83	viridiflorol		1599	1052, 2.20	-	0.12	-
84	tetradecanal		1604	1056, 1.57	-	0.03	-
85	*trans*-2-undecen-1-ol		1604	1056, 1.57	-	-	0.02
86	8,9-dehydro-neoisolongifolene		1618	1068, 2.14	0.03	0.05	-
87	humulene epoxide II		1618	1068, 2.22	0.19	0.69	-
88	unknown		1628	1076, 2.15	0.60	-	-
89	cubenol		1632	1080, 2.03	-	0.57	-
90	ledene oxide-(II)		1637	1084, 2.22	0.16	-	-
91	longipinene epoxide		1642	1088, 2.21	-	0.46	-
92	α-cadinol		1661	1104, 2.13	0.10	0.19	0.04
93	unknown		1665	1108, 2.22	-	0.59	0.10
94	bisabolol		1670	1112, 1.91	-	0.02	-
95	*trans*-farnesol		1708	1144, 1.91	0.02	0.03	-
96	unknown		1722	1156, 2.31	1.30	1.29	0.02
		Total			92.33	90.57	94.98

^1^ Rt, retention time in first dimension (s) and retention time in second dimension (s); ^2^ tr (trace), relative content <0.02%; ^3^ -, not detected. RI, the retention indices were determined in relation to a homologous series of alkanes (C_7_–C_30_) under the same operating conditions.

**Table 2:** Contact toxicity of essential oils of *C. camphora* against cotton aphids.

Samples	24 h					48 h		
	LC_50_ (mg/L)	95% CI (mg/L)	Slope ± SE	Chi Square (χ^2^)	LC_50_ (mg/L)	95% CI (mg/L)	Slope ± SE	Chi Square (χ^2^)
Leaves	312.42	249.93–376.41	1.58 ± 0.16	1.48	245.79	191.31–299.85	1.61 ± 0.16	2.97
Twigs	376.77	283.19–476.81	1.15 ± 0.14	0.56	274.99	194.13–356.11	1.13 ± 0.15	0.24
Seeds	200.92	128.05–272.58	1.08 ± 0.15	0.48	146.78	88.77–206.14	1.13 ± 0.16	0.37
Linalool	523.66	417.69–673.64	1.09 ± 0.11	1.98	262.77	202.20–333.83	1.02 ± 0.11	2.19
Imidacloprid	12.53	10.10–15.46	1.17 ± 0.10	1.17	3.58	2.84–4.39	1.45 ± 0.12	1.72

95% CI: 95% confidence interval for each LC_50_ value.

**Table 3:** Repellent rate for *C. camphora* essential oils and linalool against cotton aphids at different concentrations after 12 h and 24 h of treatment.

Time (h)	Concentration (μL/mL)	Repellent Rate (%)				Concentration (μL/mL)	Repellent Rate (%)
		Leaves	Twigs	Seeds	Linalool		DEET
12	20	75.44 ± 1.76a	70.97 ± 2.79a	76.19 ± 2.75a	/	10	88.71 ± 4.27a
	10	47.37 ± 3.03b	51.61 ± 2.79b	53.40 ± 3.17b	70.18 ± 3.51a	5	77.42 ± 4.41ab
	5	31.58 ± 5.26c	30.65 ± 1.61c	36.51 ± 1.59c	56.14 ± 4.64b	2	72.58 ± 4.34b
	2	19.30 ± 1.75d	19.35 ± 1.62d	20.63 ± 4.20c	49.12 ± 4.34bc	0.5	54.84 ± 4.63c
	1	10.53 ± 3.04d	6.45 ± 1.61e	12.70 ± 1.59d	36.84 ± 3.83cd	0.1	38.71 ± 4.27d
	0.5	/ ^1^	/	/	26.32 ± 3.04d	0.01	20.97 ± 1.61e
24	20	83.83 ± 1.47a	72.13 ± 1.64a	89.86 ± 1.45a	/	10	80.70 ± 1.75a
	10	60.29 ± 4.41b	59.01 ± 5.91b	69.57 ± 2.51b	76.56 ± 2.70a	5	71.93 ± 4.64ab
	5	42.65 ± 4.31c	52.46 ± 4.34b	55.07 ± 3.83c	65.63 ± 4.14b	2	66.67 ± 1.75b
	2	25.00 ± 2.56d	36.07 ± 5.68c	36.23 ± 1.45d	59.38 ± 4.13bc	0.5	54.38 ± 3.51c
	1	17.65 ± 3.89d	29.51 ± 5.91d	21.74 ± 0e	48.44 ± 2.71d	0.1	43.86 ± 6.32c
	0.5	/	/	/	37.50 ± 1.56e	0.01	19.3 ± 3.51d

^1^ / without treatment at the given concentration; Mean (± standard error) of three replicates for each sample. Percentage values followed by the same letter are not significantly different in the same group at *p* ≤ 0.05 (Duncan’s test).
